# Supplementary material for: Insomnia and depression levels among Malaysian undergraduate students in the Faculty of Medicine and Health Sciences (FMHS), Universiti Putra Malaysia (UPM) during Movement Control Order (MCO)
Source: PLoS One. 2023 Oct 10;18(10):e0283098. doi: 10.1371/journal.pone.0283098 (PMC10564170; doi:10.1371/journal.pone.0283098)
Supplement: Attachment — Submitted filename: Response to Reviewers.pdf [file pone.0283098.s001.pdf]

13th February 2023

Academic Editor
PLOS ONE

Dear Editor,

We thank the reviewers for their generous comments on the manuscript. On behalf of all the authors, I would like to submit rebuttal letter of “Implication of Insomnia and Depression Among Malaysian Undergraduate Health Science University Students in UPM During Covid-19/Movement Control Order” study in response to academic editor comments on this study revision.

This letter will address to journal requirements (Section A) and reviewers’ comment (Section B) as stated in “PLOS ONE Decision: Revision required [PONE-D-22-29913] - [EMID:46a201b3f656365c]” email, answered according to the question order.

Section A

1. The manuscript will comply to the PLOS ONE's style requirements.
2. Additional details of participant consent were added in the manuscript (Page 7, 2nd paragraph).
3. Data from this study are available upon request, details were noted in the cover letter.
4. ORCID ID for primary author Raja Muhammad Iqbal Bin Raja Yahya ORCID ID: 0000-0002-0550-6751 and corresponding author Sharifah Sakinah Syed Alwi ORCID ID: 0000-0002-6497-706X
5. Figure were removed in the manuscript, figure 1 in page 4 were deleted (Conceptual framework) in replaced to “Population Study” level 2 heading. In note to the deletion that Figure 2 in page 8 under “Statistical analysis” level 2 heading will change to Figure 1.
6. Typing error, the origin intend was to cited Table 7 in page 21.
7. Reference list were reviewed and updated to relevant current references. However if there is an reference error please do contact us so amendment can be made.

Section B

Reviewer #1: The sample demographic is the relative representation of the student population, though the population of female student is more compared to male student it is true reflection of gender disparity in public university in Malaysia where the numbers of female student outweighs the number of male student (Tienxhi, 2017)

Tienxhi, J. Y. (2017). The Gender Gap in Malaysian Public Universities: Examining the'Lost Boys. Journal of International and Comparative Education (JICE), 1-16.

Moreover, Inter-rater reliability was noted in the manuscript within the methodology section (Page 6, 5th paragraph).

Reviewer #2: Amendment were made based on the comments.

Reviewer #3: Differentiation level of depression and insomnia data, pre covid and post covid for these populations were not perform in this study as it only measure depression and insomnia level during the movement control order/covid-19 pandemic.

Reviewer #4: Amendment were made based on the comments.

For comment No.4 'mild/ moderate insomnia' were not used because the classification of insomnia are based from the classification from the origin paper from Morin et al., 2011.

> Morin CM, Belleville G, Bélanger L, Ivers H. The insomnia severity index: Psychometric Indicators to detect insomnia cases and evaluate treatment response. Sleep. 2011;34(5):601–8.

For comment No.5 the reason why, the rates were compared study done outside Malaysia because during the time of writing there are limited studies in Malaysia that addressing depression and insomnia in university students. Moreover, rates comparison to Sundarasen et al., 2020 were unable to be made because the paper focuses on the anxiety levels of student during the lockdown.

For comment No.7 rumination was discussed as a separate entity for theoretical or conceptual reasons, or to provide background or context for the study.

In conclusion, I have presented clear evidence and arguments refuting the claims made by the reviewers in their evaluation of our paper. I believe that our research is scientifically sound and deserves to be published in PLOS ONE. I hope that after reviewing my rebuttal, the publisher will recognize the validity of our findings and make the necessary corrections to the paper.

Thank you for considering this study and I look forward to your positive response.

Sincerely,
Raja Muhammad Iqbal Bin Raja Yahya
